# Free surface aeration and development dependence in chute flows

**DOI:** 10.1038/s41598-022-05588-y

**Published:** 2022-01-27

**Authors:** Wangru Wei, Jun Deng

**Affiliations:** State Key Lab of Hydraulics and Mountain River Engineering, Sichuan University, Chengdu, 610065 China

**Keywords:** Engineering, Physics

## Abstract

The microscopic description of the mixture behaviour of air–water flow remains a challenge. It is not clear how to represent a complex two-phase interaction with the turbulent air–water structure development process. In this study, based on the air–water mixing fluctuation properties in self-aerated chute flows, a prediction model of air concentration distribution related to a theoretical transition depth is developed. The air–water turbulent mixing analysis reveals that the mixture flow depth, at which the local air concentration is 0.5, represents the interior transition boundary. The agreement of the calculated results with the test data confirms that local water and air turbulent mixing through the free surface area should be equally considered. On the basis of the interior transition depth, the development of self-aeration mainly manifests as the air turbulent mixing process in the low aerated region, while the water turbulent mixing in the high aerated region remains mostly unchanged. A series of relationships concerning the development of self-aerated flows is proposed to enable quantitative estimations in practical applications of water engineering.

Owing to the high turbulence intensity of free surface flows (Fig. 1), water droplets and air bubbles are key flow structures in self-aerated chute flows, and both air–water mixtures and development processes are important considerations in engineering applications. Understanding the transport mechanisms that govern air–water flow in water release structures has direct implications for natural and industrial processes^1,2^. For air–water mixture flow bulking, the average elevation where the expelled water droplets can reach is of prime importance^3^. For cavitation erosion protection in high-speed open channel flows and air–water transfer of atmospheric gases in natural streams or conduits, the air bubble concentration distribution is of a higher significance because of the net air–water interface area of thousands of tiny bubbles^4,5^. Adequate management of the total dissolved gas requires an accurate prediction of air–water mixture development within the concentration distribution to meet ecosystem demands. Thus, the development of air–water diffusion in high-speed chute flows has received significant attention.

**Figure 1:**
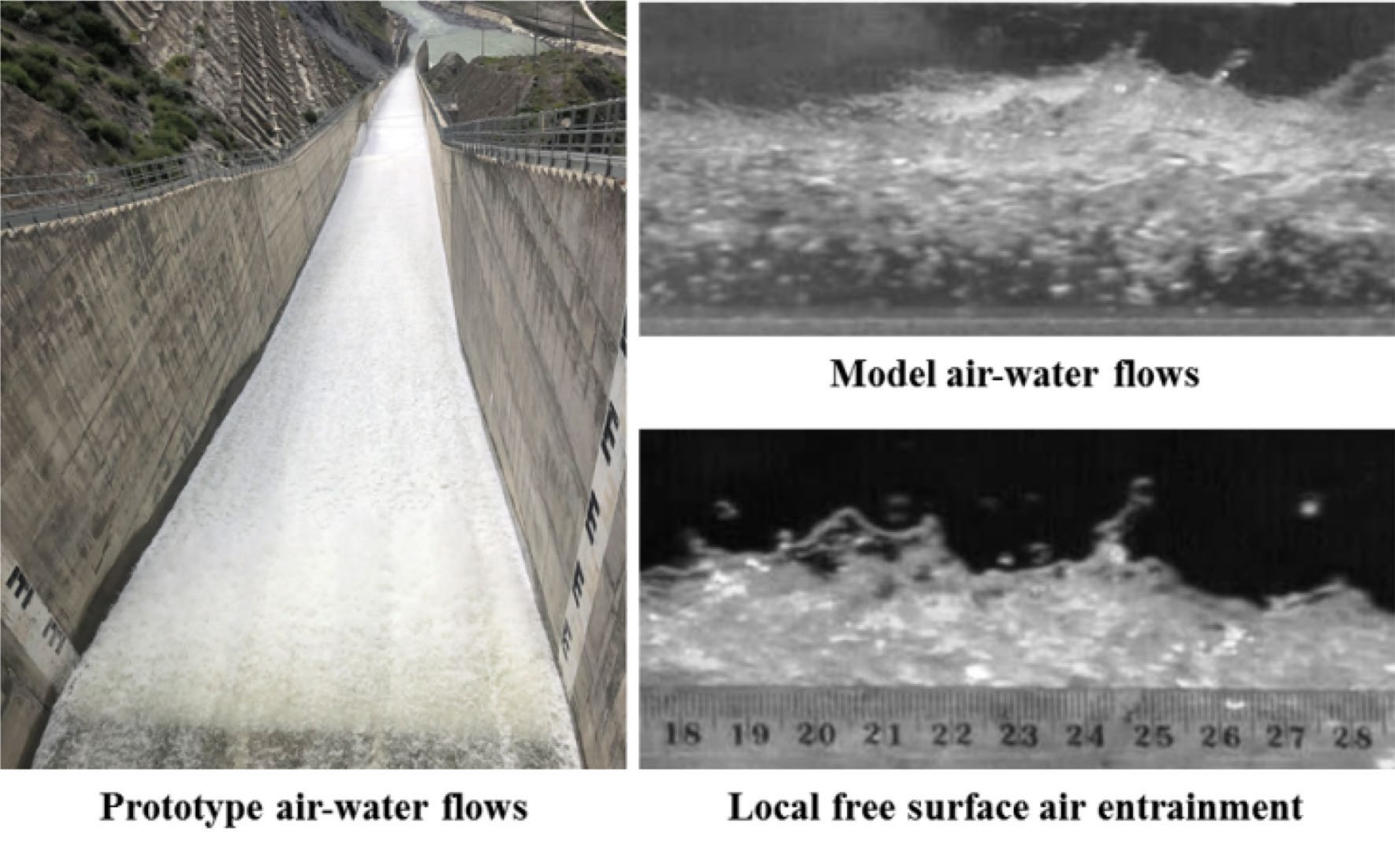
Typical air–water patterns of self-aerated flows in prototype spillways and laboratory models.

Air–water mixtures were recognized to be a result of their interaction under the turbulence effect. Some similarities between air bubbles and water droplets were observed for a water jet discharging into the atmosphere. Detailed hydrodynamic analyses on the interaction of the air–water surface resulted in a critical condition of free-surface perturbation breakup as a criterion for aeration onset^6^. For engineering purposes, the air–water mixture intensity was commonly described as the air concentration *C*_a_, which was the ratio of air volume to the sum of the air and water volumes. The characterized flow depth *y*_90_, where the local *C*_a_ = 0.9 at the flow cross-section, was widely used as the mixture flow depth to refer to the overall bulk^7,8^. The mixture of water and air parcels resulted in a profile of air concentration distribution in which the air concentration increased from a minimum value at the channel bottom to the free surface. A typical approach for cross-sectional air concentration distributions was an advective–diffusive theory based on air–water mixture levels^9,10^. Valero and Bung^11^ extended the analytical application for the air concentration distribution considering outer wave interface effects. Based on air concentration measurements and flow pattern observations of self-aerated open channel flows, researchers analysed the cross-sectional mixture flow on the basis of “layers”, such as individual water droplets in air at the upper layer and individual air bubbles in water at the lower layer^12,13^. The transition depth of two layers was defined for which d^2^*C*_a_/d*y*^2^ = 0 to develop a model to predict air concentration distributions^7^. However, an estimate for the specific depth was not provided, which was affected by several hydraulic conditions. Using a turbulent transport process analysis, the air distribution was logarithmic in the lower layer and was more closely approximated by a cumulative log-normal profile in the upper layer. The conservation equation for the air concentration distribution was developed by characterizing the parcel movement velocity of air bubbles over the entire flow depth^10^.

Recently, as measurement and analysis methods for air–water chute flows developed, more detailed characteristics of the mixture flow were investigated^14–16^. For turbulent air–water flows in open channels, the air concentration fluctuation caused by the velocity fluctuation was considered in the air bubble diffusion process of air–water flows. Based on detailed interactions of bubbles with local water in the lower region where *C*_a_ < 0.30 and the substantial ejections of water droplets in the upper region where *C*_a_ > 0.70, the intrusive phase-detection measurements highlighted the strong air–water turbulent interactions in the intermediate cross-sectional area where 0.30 < *C*_a_ < 0.70^17,18^. According to the definition of air concentration in air–water flows, the quantities of water and air are equal if the local *C*_a_ = 0.50. The specific flow depth *y*_50_, where *C*_a_ = 0.50, can be considered canonically to undergo a transition from bubbly flow to droplet flow in the cross-section. Basic theoretical analyses confirmed that the specific flow depth *y*_50_ can be used as a hypothesis condition to describe the air concentration distribution of self-aerated open channel flows^19,20^. It can be deduced that owing to the difference in the air–water structures at different layers of the flow cross-section, there must be an interior transition flow depth that can represent the detailed air–water structure development.

However, despite the key role of the interior transition depth in the air–water turbulent mixing process, theoretical verifications concerning the specific transition depth in self-aerated open channel flows are limited. Little information about the effect of particle turbulence on the average air concentration distribution in self-aerated flows is available. This study focuses on the air–water mixing affected by turbulence to analyse the theoretical transition depth in self-aerated chute flows. The prediction of the air concentration distribution from the transition depth represents the first attempt to verify the rationality of the interior transition depth. Moreover, this study provides further insights into air–water development differentiated by the transition depth of self-aerated flows, aiming to propose quantitative estimations in practical applications.

## Concentration and density fluctuations in turbulent air–water flows

For a time average air–water control volume, the volume of the control mixture parcel is *V*_0_, which is equal to the product of the unit area *A*_0_ and the displacement distance per unit time. The time average air and water concentrations of the control mixture parcel are defined as *C*_a0_ = *V*_a_/*V*_0_ and *C*_w0_ = *V*_w_/*V*_0_, respectively. The time average density of the control mixture volume is *ρ*_m0_. The mass of the mixture volume *M*_0_ is defined as *M*_0_ = *ρ*_m0_*V*_0_, where *V*_0_ is the time average volume equal to *V*_a_ + *V*_w_. For turbulent air–water flows, the time average parameter is a decomposition result as the sum of the time average mean factor and the time average fluctuation factor. To retain *ρ*_m0_, it is hypothesized that the value of *M*_0_ is approximately equal to

1 $$ M_{0} \approx \rho_{{{\text{m0}}}} C_{{{\text{a0}}}} V_{{0}} + \rho_{{{\text{m0}}}} C_{{{\text{w0}}}} V_{{0}} $$

The two terms can be considered as the mass rate of air contribution *M*_a_ = *ρ*_m0_*C*_a0_*V*_0_ and mass rate of water contribution *M*_w_ = *ρ*_m0_*C*_w0_*V*_0_. In self-aerated open channel flows, there are two basic transportation patterns in the *y*-direction perpendicular to the streamwise direction, that is, the air phase and water phase transportations. If it is considered that the mixture fluid crosses the unit area per time, the two terms become *ρ*_m0_*C*_a0_*u*_a_^*^ and *ρ*_m0_*C*_w0_*u*_w_^*^, where *u*_a_^*^ and *u*_w_^*^ are two characteristic velocities, representing the transition of the control volume. They consist of the turbulent eddy fluctuation velocity *u*_*y*_*'*, droplet falling-back velocity *u*_f_ and air bubble rising velocity *u*_r_.

The definition of the characterized air–water volume is related to the fully developed two-phase flow on the basis of the mixing length theory^21^. The air–water transfer occurs under a uniform air–water mixture, and the air–water flow behaves as a homogeneous mixture across the flow section^22,23^. This ensures sufficient time-average air–water control volumes for flow property analyses. Both of them are the interaction of turbulence fluctuation and gravity or buoyancy forces, resulting in volume concentration and density changes. The time average fluctuation air concentration shows an important role in drag reduction, and the production of a negative turbulent shear stress component is obtained using momentum conservation analysis^24,25^. This indicates that the air bubble interfacial transfer for the unit air–water control volume requires vital attention and that the time-average fluctuation air concentration affected by the fluctuation velocity can be used as a component part of the turbulent air–water control volume. For flow density properties, the mass transfer among air–water control particles is the accompanying appearance causing the time average fluctuation of unit volume density. The mass transfer intensity is significantly correlated with local eddy shear, fluctuation, bubble and air–water surface transfer effects in open channel turbulent flows^26–29^. For an average air–water control volume with a constant concentration, more bubbles with smaller size can significantly raise the specific surface area, compared to less bubbles with larger bubble size dominant condition. General turbulent models are established based on the microscopic air–water mass transfer and bubble density and size variations^30–32^. Thus, the density variation of the control volume should be considered due to microscopic air–water structures. In the present study, the deviation of the local time average mean density of air–water control particles is the time average fluctuation of particle density. The effect of the time average fluctuation of particle density is among air–water control particles, and the entire air–water mass is constant with the conservation of mass. The time-average fluctuation density affected by the flow turbulence and fluctuation factors can be used as a component part of the turbulent air–water control volume. Consequently, for air and water diffusion generalizations, the approach of mean and fluctuating decompositions for the air concentration and mixture density is used in turbulent self-aerated chute flows.

**Figure 2:**
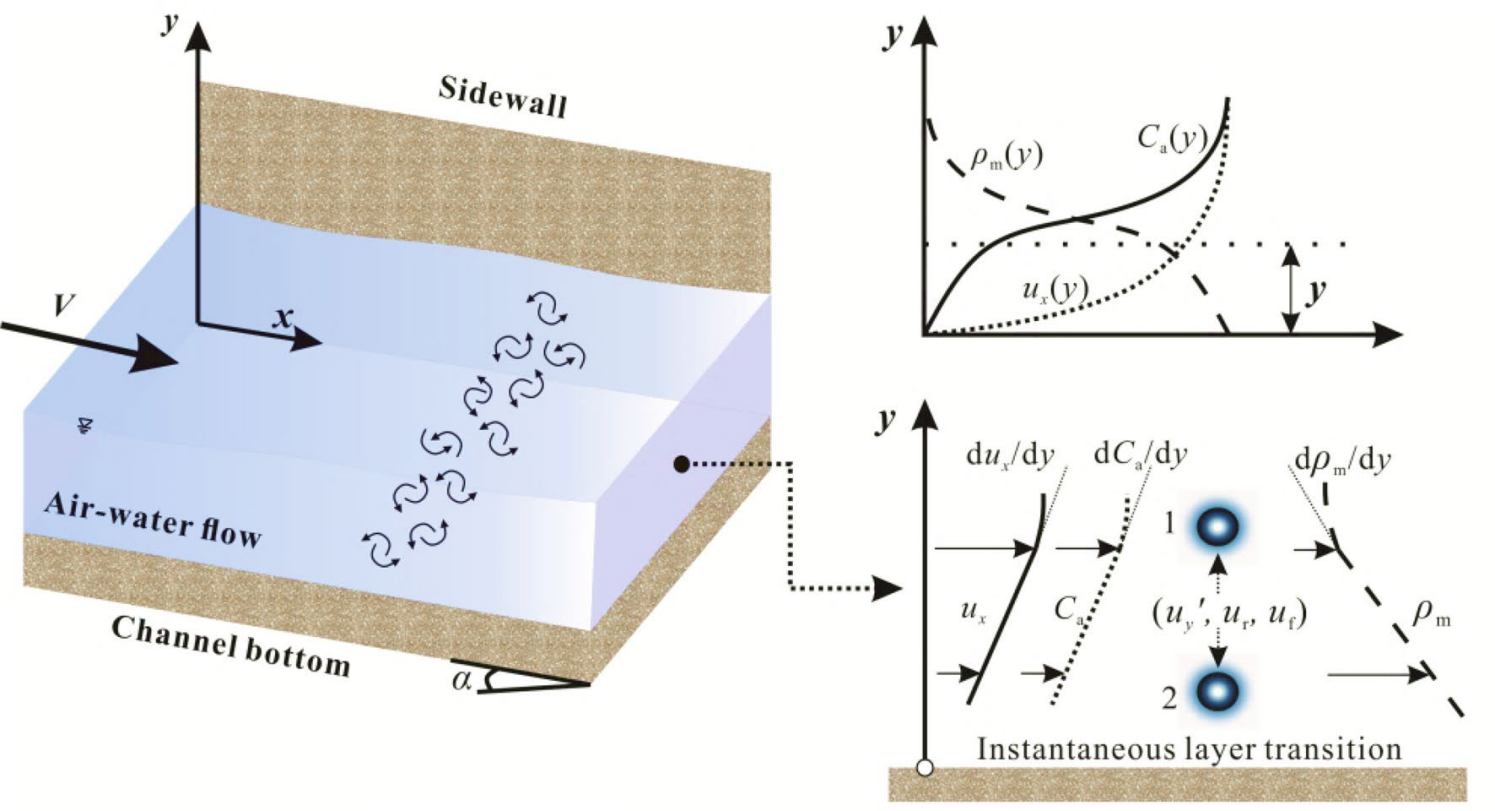
Schematic definitions for the turbulence fluctuations of air–water mixture parcels (*α* is the channel slope).

Prandtl’s mixing length theory is introduced to hypothesize the time-average mean and fluctuation for concentration and density properties, which are determined by the exchange on a macroscale analogous to that of molecular motion^21^. Figure 2 schematically shows an air–water mixture volume transition between layer 1 and layer 2 affected by the characteristic velocities. For example, due to *u*_*y*_′ in the − *y* direction, the density and air concentration of the air–water mixture volume at level 1 are retained when it just arrives at level 2, and the density and air concentration at level 2 decrease soon after by exchanging with the flow in the neighbourhood of level 2. The detailed air–water property fluctuations are shown in Table 1, where the time-average and fluctuation local densities are *ρ*_m_ and *ρ*_m_*'*; the time-average mean and fluctuation water droplet concentration and *C*_w_ and *C*_w_*'*; the time-average mean and fluctuation air bubble concentration are *C*_a_ and *C*_a_*'*. In the vertical direction (*y*), the air–water mixture volume is presumed to retain its original time-averaged properties at the arrival position, including velocity, density, and concentration^33–35^. Thus, the vertical density and air concentration fluctuations of the air–water mixture parcel through level 1 are given by *ρ*_m_′ < 0 and *C*_a_′ > 0, respectively. Based on these definitions for density and air concentration fluctuations of air–water mixture volumes, it is deduced that both the density and air concentration fluctuations are caused by the turbulent motion of mixture parcels. For other factors, *u*_f_ and *u*_r_, there are different exchange motion processes analogous to the turbulent eddy effect. The relationship between the time-average concentration and mixture flow density^7^ is *ρ*_m0_ ≈ *ρ*_w0_ (1 − *C*_a0_) = *ρ*_w0_*C*_w0_, where *ρ*_w0_ is the water density. Considering this related expression, the fluctuation density of the air–water mixture volume is

2 $$ \rho_{{\text{m}}}^{\prime } = \rho_{{\text{w}}} (1 - C_{{{\text{a0}}}} ) - \rho_{{\text{w}}} (1 - C_{{\text{a}}} ) = - \rho_{{\text{w}}} C_{{\text{a}}}^{\prime } $$

**Table 1:** Fluctuation properties in the air–water mixing process.

Parameters	Density		Concentration	
*u*_*y*_′ > 0	*ρ*_m_′ > 0	*ρ*_m0_ = *ρ*_m_ + *ρ*_m_′	*C*_w_′ > 0	*C*_w0_ = *C*_w_ + *C*_w_′
*u*_*y*_′ < 0	*ρ*_m_′ < 0	*ρ*_m0_ = *ρ*_m_ − *ρ*_m_′	*C*_a_′ > 0	*C*_a0_ = *C*_a_ + *C*_a_′
*u*_r_ > 0	*ρ*_m_′ > 0	*ρ*_m0_ = *ρ*_m_ + *ρ*_m_′	*C*_a_′ < 0	*C*_a0_ = *C*_a_ − *C*_a_′
*u*_f_ < 0	*ρ*_m_′ < 0	*ρ*_m0_ = *ρ*_m_ − *ρ*_m_′	*C*_w_′ < 0	*C*_w0_ = *C*_w_ − *C*_w_′

In the present study, the mixture of air and water can be approximated as two diffusion processes, that is, the air mixing process and water mixing process. Thus, the time-average mean and fluctuation for concentration and density properties are treated, respectively.

All results are presented at normal temperatures and standard atmospheric pressure conditions. The free surface aeration and air–water mixture processes are determined by the free surface air entrainment capacity and flow turbulence intensity. Uniform equilibrium air–water flows are defined as the air concentration distribution independent of the streamwise direction. The fully aeration condition means the cross-sectional fully air diffusion across the flow section without the clear water layer existence. Detailed descriptions of the two turbulent mixing processes combined with multiple fluctuation properties are shown in the following sections.

## Air turbulent mixing

The time average mass flux of air contribution *m*_a1_ per unit area transported by the local turbulent fluctuation *u*_*y*1_′ in the − *y*-direction is

3 $$ m_{a1} = {(}\rho_{{\text{m}}} - \rho_{{\text{m}}}^{\prime } ) \cdot {(}C_{{\text{a}}} { + }C_{{\text{a}}}^{\prime } ) \cdot u_{y1}^{\prime } = \rho_{{\text{m}}} C_{{\text{a}}} u_{y1}^{\prime } { + }\rho_{{\text{m}}} C_{{\text{a}}}^{\prime } u_{y1}^{\prime } - C_{{\text{a}}} \rho_{{\text{m}}}^{\prime } u_{y1}^{\prime } - \rho_{{\text{m}}}^{\prime } C_{{\text{a}}}^{\prime } u_{y1}^{\prime } $$

The terms *C*_a_*'u*_*y*1_′ and *ρ*_m_*'u*_*y*1_′ are considered turbulent diffusion rates affected by the air phase. The turbulent diffusion processes for both the air concentration and mixture density are identical, which are analogous to the Boussinesq hypothesis^31^. They are proportional to the gradient of the time-average concentration, given by

4 $$ C_{{\text{a}}}^{\prime } u_{y1}^{\prime } = K_{{{\text{a1}}}} \frac{{{\text{d}}C_{{\text{a}}} }}{{{\text{d}}y}} $$

5 $$ \rho_{{\text{m}}}^{\prime } u_{y1}^{\prime } = - K_{{{\text{a1}}}} \frac{{{\text{d}}\rho_{{\text{m}}} }}{{{\text{d}}y}} $$

where *K*_a1_ is a characterized turbulent diffusion coefficient. For steady flow in an open channel, Eq. (3) yields

6 $$ \overline{{m_{a1} }} = \rho_{{\text{m}}} K_{{{\text{a1}}}} \frac{{{\text{d}}C_{{\text{a}}} }}{{{\text{d}}y}} + C_{{\text{a}}} K_{{{\text{a1}}}} \frac{{{\text{d}}\rho_{{\text{m}}} }}{{{\text{d}}y}} - \overline{{\rho_{{\text{m}}}^{\prime } C_{{\text{a}}}^{\prime } u_{y1}^{\prime } }} = {(1} - 2C_{{\text{a}}} )\rho_{{\text{w}}} K_{{{\text{a1}}}} \frac{{{\text{d}}C_{{\text{a}}} }}{{{\text{d}}y}} - \overline{{\rho_{{\text{m}}}^{\prime } C_{{\text{a}}}^{\prime } u_{y1}^{\prime } }} $$

The time average mass flux of air contribution *m*_a2_ rising up owing to the buoyancy effect *u*_r_ in the + *y*-direction is

7 $$ m_{a2} { = (}\rho_{{\text{m}}} + \rho_{{\text{m}}}^{\prime } ) \cdot {(}C_{{\text{a}}} - C_{{\text{a}}}^{\prime } ) \cdot u_{{\text{r}}} {\text{cos}}\alpha { = }\rho_{{\text{m}}} C_{{\text{a}}} u_{{\text{r}}} {\text{cos}}\alpha - \rho_{{\text{m}}} C_{{\text{a}}}^{\prime } u_{{\text{r}}} {\text{cos}}\alpha + C_{{\text{a}}} \rho_{{\text{m}}}^{\prime } u_{{\text{r}}} {\text{cos}}\alpha - \rho_{{\text{m}}}^{\prime } C_{{\text{a}}}^{\prime } u_{{\text{r}}} {\text{cos}}\alpha $$

The terms *C*_a_*'u*_r_cos*α* and *ρ*_m_*'u*_r_cos*α* are considered the diffusion rates by the buoyancy effect, given by

8 $$ C_{{\text{a}}}^{\prime } u_{{\text{r}}} {\text{cos}}\alpha { = }K_{{{\text{a2}}}} \frac{{{\text{d}}C_{{\text{a}}} }}{{{\text{d}}y}} $$

9 $$ \rho_{{\text{m}}}^{\prime } u_{{\text{r}}} {\text{cos}}\alpha { = } - K_{{{\text{a2}}}} \frac{{{\text{d}}\rho_{{\text{m}}} }}{{{\text{d}}y}} $$

where *K*_a2_ is the diffusion coefficient affected by the buoyancy. For steady flow in an open channel, Eq. (7) yields

10 $$ \overline{{m_{a2} }} { = }\rho_{{\text{w}}} (1 - C_{{\text{a}}} )C_{{\text{a}}} u_{{\text{r}}} {\text{cos}}\alpha - \rho_{{\text{w}}} K_{{{\text{a2}}}} (1 - 2C_{{\text{a}}} )\frac{{{\text{d}}C_{{\text{a}}} }}{{{\text{d}}y}} - \overline{{\rho_{{\text{m}}}^{\prime } C_{{\text{a}}}^{\prime } }} u_{{\text{r}}} {\text{cos}}\alpha $$

For uniform mixing of air in the water, the mass flux of air entrained in the − *y*-direction equals that of air rising in the + *y*-direction, that is,

11 $$ \overline{{m_{a1} }} { = }\overline{{m_{a2} }} $$

Then, it is obtained as

12 $$ {(1} - 2C_{{\text{a}}} )(K_{{{\text{a1}}}} + K_{{{\text{a2}}}} )\frac{{{\text{d}}C_{{\text{a}}} }}{{{\text{d}}y}} = (1 - C_{{\text{a}}} )C_{{\text{a}}} u_{{\text{r}}} {\text{cos}}\alpha + \frac{{\overline{{\rho_{{\text{m}}}^{\prime } C_{{\text{a}}}^{\prime } u_{y1}^{\prime } }} - \overline{{\rho_{{\text{m}}}^{\prime } C_{{\text{a}}}^{\prime } }} u_{{\text{r}}} {\text{cos}}\alpha }}{{\rho_{{\text{w}}} }} $$

Considering that the flow density fluctuation *ρ*_m_*'* is much smaller than the water density *ρ*_w_, that is, *ρ*_m_*'* <  < *ρ*_w_, and the higher order of multiple *ρ*_m_*'C*_a_*'u*_*y*_*'* and *ρ*_m_*'C*_a_*'u*_r_cos*α*, the second term on the right term is neglected, that is,

13 $$ {(1} - 2C_{{\text{a}}} )(K_{{{\text{a1}}}} + K_{{{\text{a2}}}} )\frac{{{\text{d}}C_{{\text{a}}} }}{{{\text{d}}y}} = (1 - C_{{\text{a}}} )C_{{\text{a}}} u_{{\text{r}}} {\text{cos}}\alpha $$

It should be noted that in the right term, (1 − *C*_a_)*C*_a_*u*_r_cos*α* > 0, and in the left term, *K*_a1_ + *K*_a2_ > 0 and d*C*_a_/d*y* > 0. The case for which the air phase continues to diffuse during the turbulent mixing process is given by the condition that the sign of the left term is positive; that is, *C*_a_ < 0.5 should be maintained to ensure that Eq. (13) is satisfied. This indicates that based on the air mixing process under the turbulent diffusion effect, the limiting air concentration is *C*_a_ < 0.5.

The alternative formulation for the final rising velocity of air parcel under a local concentration condition is introduced: *u*_r_ = (*u*_r_)_0_(1 − *C*_a_)^2^, where (*u*_r_)_0_ is the final velocity of an individual air parcel in pure water^36^. This is a generalized expression that assumes that the control volume movement is affected by mixture flow density variation and does not interact with each other. A dimensionless air turbulent diffusivity *K*_a_ and a dimensionless distance y^*^ are normalized by a flow depth *y*_50_, where local *C*_a_ = 0.5, given as

14 $$ K_{{\text{a}}} = \frac{{y_{50} (u_{{\text{r}}} {)}_{0} {\text{cos}}\alpha }}{{K_{{{\text{a1}}}} + K_{{{\text{a2}}}} }},\quad y^{*} { = }\frac{y}{{y_{50} }} $$

Then, Eq. (13) yields

15 $$ \frac{{{1} - 2C_{{\text{a}}} }}{{C_{{\text{a}}} (1 - C_{{\text{a}}} )^{3} }}\frac{{{\text{d}}C_{{\text{a}}} }}{{{\text{d}}y^{*} }} = K_{{\text{a}}} $$

Integrating Eq. (15) in the *y*-direction yields

16 $$ \frac{1}{{1 - C_{{\text{a}}} }} - {\text{ln}}\frac{{1 - C_{{\text{a}}} }}{{C_{{\text{a}}} }} - \frac{1}{{2(1 - C_{{\text{a}}} )^{2} }} = K_{{\text{a}}} y^{*} + D_{{\text{a}}} $$

where *D*_a_ is the integration constant. This relation is a function of the air concentration distribution in the region where *C*_a_ ≤ 0.5. The relationship between *K*_a_ and *D*_a_ can be deduced from *C*_a_ = 0.5 for y^*^ = 1, that is,

17 $$ K_{{\text{a}}} + D_{{\text{a}}} = 0 $$

## Water turbulent mixing

The time average mass flux of water contribution *m*_w1_ per unit area transported by the local turbulent fluctuation *u*_*y*2_′ in the + *y*-direction is

18 $$ m_{{{\text{ w}}1}} = {(}\rho_{{\text{m}}} + \rho_{{\text{m}}}^{\prime } ) \cdot {(}C_{{\text{w}}} { + }C_{{\text{w}}}^{\prime } ) \cdot u_{y2}^{\prime } { = }\rho_{{\text{m}}} C_{{\text{w}}} u_{y2}^{\prime } { + }\rho_{{\text{m}}} C_{{\text{w}}}^{\prime } u_{y2}^{\prime } + C_{{\text{w}}} \rho_{{\text{m}}}^{\prime } u_{y2}^{\prime } + \rho_{{\text{m}}}^{\prime } C_{{\text{w}}}^{\prime } u_{y2}^{\prime } $$

The terms *C*_w_*'u*_*y*2_*'* and *ρ*_m_*'u*_*y*2_*'* are considered the turbulent diffusion rate affected by the water phase. The turbulent diffusion processes for both the water concentration and mixture density are identical, and they are proportional to the gradient of the time-average concentration, given by

19 $$ C_{{\text{w}}}^{\prime } u_{y2}^{\prime } = - K_{{{\text{w1}}}} \frac{{{\text{d}}C_{{\text{w}}} }}{{{\text{d}}y}} $$

20 $$ \rho_{{\text{m}}}^{\prime } u_{y2}^{\prime } = - K_{{{\text{w1}}}} \frac{{{\text{d}}\rho_{{\text{m}}} }}{{{\text{d}}y}} $$

where *K*_w1_ is a turbulent diffusion coefficient. For steady flow in an open channel, Eq. (18) yields

21 $$ \overline{{m_{{{\text{ w}}1}} }} = - 2\rho_{{\text{w}}} C_{{\text{w}}} K_{{{\text{w1}}}} \frac{{{\text{d}}C_{{\text{w}}} }}{{{\text{d}}y}} + \overline{{\rho_{{\text{m}}}^{\prime } C_{{\text{a}}}^{\prime } u_{y2}^{\prime } }} $$

The time average mass flux of water contribution *m*_w2_ falling back owing to the gravity effect in the − *y*-direction *u*_f_ is

22 $$ m_{{{\text{w}}2}}^{\prime } = {(}\rho_{{\text{m}}} - \rho_{{\text{m}}}^{\prime } ) \cdot {(}C_{{\text{w}}} - C_{{\text{w}}}^{\prime } ) \cdot u_{{\text{f}}} \cos \alpha = \rho_{{\text{m}}} C_{{\text{w}}} u_{{\text{f}}} \cos \alpha - \rho_{{\text{m}}} C_{{\text{w}}}^{\prime } u_{{\text{f}}} \cos \alpha - C_{{\text{w}}} \rho_{{\text{m}}}^{\prime } u_{{\text{f}}} \cos \alpha + \rho_{{\text{m}}}^{\prime } C_{{\text{w}}}^{\prime } u_{{\text{f}}} \cos \alpha $$

The terms *C*_w_*'u*_f_cos*α* and *ρ*_m_*'u*_f_cos*α* are considered the diffusion rates by the gravity effect, given by

23 $$ C_{{\text{w}}}^{\prime } u_{{\text{f}}} \cos \alpha = - K_{{{\text{w2}}}} \frac{{{\text{d}}C_{{\text{w}}} }}{{{\text{d}}y}} $$

24 $$ \rho_{{\text{m}}}^{\prime } u_{{\text{f}}} \cos \alpha = - K_{{{\text{w2}}}} \frac{{{\text{d}}\rho_{{\text{m}}} }}{{{\text{d}}y}} $$

where *K*_w2_ is a diffusion coefficient affected by gravity. For steady flow in an open channel, Eq. (22) yields

25 $$ \overline{{m_{{{\text{w}}2}} }} = C_{{\text{w}}}^{2} \rho_{{\text{w}}} u_{{\text{f}}} \cos \alpha + 2\rho_{{\text{w}}} C_{{\text{w}}} K_{{{\text{2w}}}} \frac{{{\text{d}}C_{{\text{w}}} }}{{{\text{d}}y}} + \overline{{\rho_{{\text{m}}}^{\prime } C_{{\text{w}}}^{\prime } }} u_{{\text{f}}} \cos \alpha $$

For uniform mixing of water in the air, the mass flux of the water contribution expelled in the + *y*-direction equals that falling back in the − *y*-direction, that is,

26 $$ \overline{{m_{{{\text{w}}1}} }} { = }\overline{{m_{{{\text{w}}2}} }} $$

Then, it is obtained as

27 $$ - 2C_{{\text{w}}} (K_{{{\text{w1}}}} + K_{{{\text{2w}}}} )\frac{{{\text{d}}C_{{\text{w}}} }}{{{\text{d}}y}} = C_{{\text{w}}}^{2} u_{{\text{f}}} \cos \alpha + \frac{{\overline{{\rho_{{\text{m}}}^{\prime } C_{{\text{w}}}^{\prime } }} u_{{\text{f}}} \cos \alpha - \overline{{\rho_{{\text{m}}}^{\prime } C_{{\text{a}}}^{\prime } u_{y2}^{\prime } }} }}{{\rho_{{\text{w}}} }} $$

This is analogous to the previous analysis; the second term on the right term is neglected, that is,

28 $$ - 2C_{{\text{w}}} (K_{{{\text{w1}}}} + K_{{{\text{2w}}}} )\frac{{{\text{d}}C_{{\text{w}}} }}{{{\text{d}}y}} = C_{{\text{w}}}^{2} u_{{\text{f}}} \cos \alpha $$

The alternative velocity formulation for the local concentration condition in the mixture fluid is introduced as *u*_f_ = (*u*_f_)_0_(1 − *C*_w_)^2^, where (*u*_f_)_0_ is the final velocity of an individual water parcel. A dimensionless turbulent diffusivity *K*_w_ is defined as

29 $$ K_{{\text{w}}} { = }\frac{{y_{50} (u_{{\text{f}}} {)}_{0} {\text{cos}}\alpha }}{{K_{{{\text{w1}}}} + K_{{{\text{w2}}}} }} $$

Then, Eq. (28) with the dimensionless y^*^ yields

30 $$ \frac{ - 2}{{C_{{\text{w}}} (1 - C_{{\text{w}}} )^{2} }}\frac{{{\text{d}}C_{{\text{w}}} }}{{{\text{d}}y^{*} }} = K_{{\text{w}}} $$

Integrating Eq. (30) in the *y*-direction yields

31 $$ 2\left( {\ln \frac{{1 - C_{{\text{w}}} }}{{C_{{\text{w}}} }} - \frac{1}{{1 - C_{{\text{w}}} }}} \right) = K_{{\text{w}}} y^{*} + D_{{\text{w}}} $$

where *D*_w_ is the integration constant. This relation is a function of the water concentration distribution. The air concentration distribution can be obtained by *C*_a_ = 1 − *C*_w_, that is,

32 $$ 2\left( {\ln \frac{{C_{{\text{a}}} }}{{1 - C_{{\text{a}}} }} - \frac{1}{{C_{{\text{a}}} }}} \right) = K_{{\text{w}}} y^{*} + D_{{\text{w}}} $$

Considering *y*_90_ as the total mixture flow depth where the local water droplet concentration is *C*_w_ = 0.1 (and *C*_a_ = 0.9), the relationship between *K*_w_ and *D*_w_ can be deduced under two boundary conditions: *C*_w_ = 0.5 for y^*^ = 1 and *C*_w_ = 0.1 for y^*^ = *y*_90_/*y*_50_, which yields

33 $$ K_{{\text{w}}} { = }\frac{6.172}{{y_{90} /y_{50} - 1}},\quad D_{{\text{w}}} { = } - 4 - K_{{\text{w}}} $$

Comparing the air and water turbulent mixing Eqs. (15) and (30), it is found that the concentration gradient variation is continuous for the water phase, while it is not continuous for the air phase in air–water flows. Thus, this theoretical analysis reveals that *y*_50_, where *C*_a_ = 0.5, represents the interior boundary for air turbulent mixing and water turbulent mixing in which the water phase can be considered a homogenous phase.

## Air concentration distributions

The turbulent diffusion coefficients *K*_a_ and *K*_w_ are determined by the relationship between the aeration level and *y*_50_/*y*_90_. The average cross-sectional air concentration *C*_mean_ is defined as the integration of the local *C*_a_ over the mixture flow depth between the channel bottom at *y* = 0 and *y*_90_:

34 $$ C_{{{\text{mean}}}} = \frac{1}{{y_{90} }}\int\limits_{y = 0}^{{y = y_{90} }} {C(y)} {\text{d}}y $$

**Figure 3:**
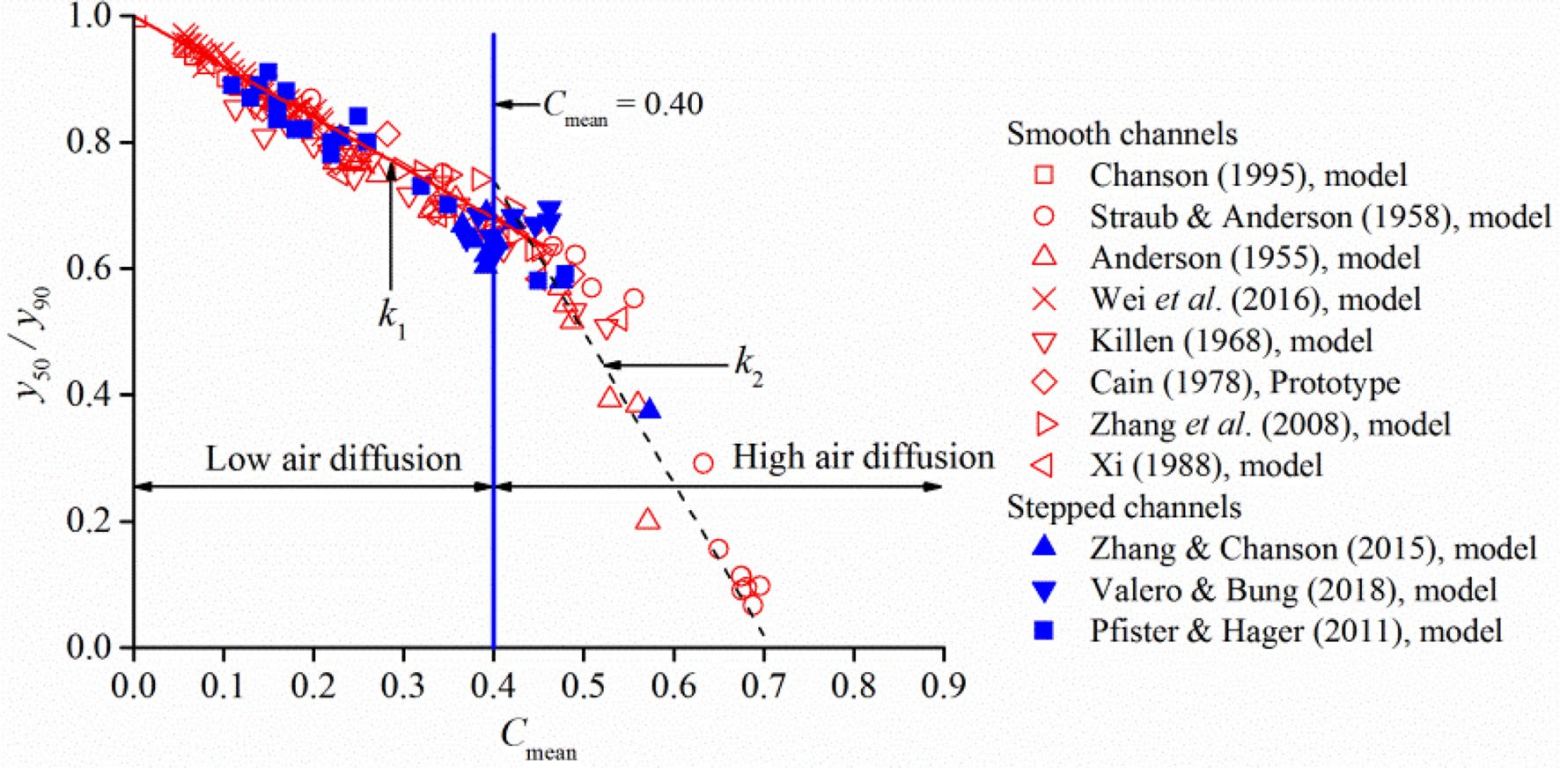
The variation of *y*_50_/*y*_90_ with the increase of *C*_mean_ in self-aerated open channel flows.

The statistical relationships between *y*_50_/*y*_90_ and *C*_mean_ are shown in Fig. 3 for different model and prototype chute flows. For a constant channel slope condition, *y*_50_/*y*_90_ decreases with increasing *C*_mean_, and it is independent of other hydraulic conditions, such as approach flow conditions, smooth or stepped channel type, and slope. For *C*_mean_ values smaller than approximately 0.40, a linear trend is followed with a constant gradient *k*_1_ ≈ − 0.8. The gradient changes to *k*_2_ ≈ − 2.4 for *C*_mean_ greater than approximately 0.40, for which the mixture flow cross-section is fully aerated and the local air concentration is greater than 0.10. On the basis of *C*_mean_ = 0.40, the two relationships can be expressed as *y*_50_/*y*_90_ = 1–0.8*C*_mean_ for *C*_mean_ < 0.40 and *y*_50_/*y*_90_ = 1.7–2.4*C*_mean_ for *C*_mean_ > 0.40. This indicates that the interior transition boundary of turbulence diffusion changes suddenly if the aeration development of the flow cross-section exceeds *C*_mean_ = 0.40.

**Figure 4:**
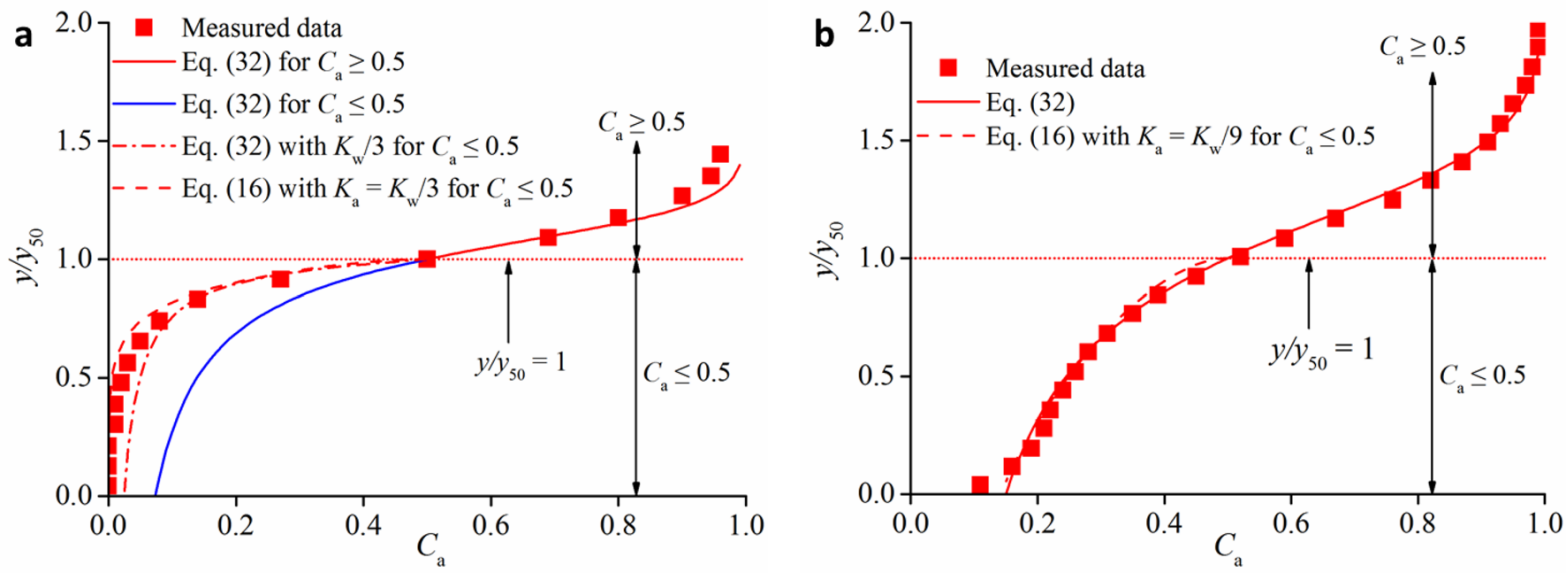
Air concentration distribution for fully and partially aerated diffusions: (**a**) *C*_mean_ = 0.23, (**b**) *C*_mean_ = 0.43.

As aeration develops through the turbulent free surface, the air–water mixing layer diffuses into the water flow, protruding to the channel bottom. For some cases, the entire flow is partially aerated, where a clear water layer exists at the bottom area of the entire flow. For the other cases, the entire flow section becomes fully aerated without the presence of clear water. Here, the air concentration distributions obtained from the prototype Aviemore dam spillway^37^ are used for detailed analysis. For partially aerated flows, the measured data showed that the local *C*_a_ at the bottom area was mostly smaller than 0.05 for *C*_mean_ < 0.40. If *K*_w_ and *D*_w_ are obtained from Eq. (33), the comparison between the measured data and Eq. (32) in Fig. 4a shows that the theoretical prediction is acceptable only for *C*_a_ > 0.5, while for *C*_a_ < 0.5, Eq. (32) overestimates the air diffusion in the low aerated region. This indicates that the presence of a clear water layer can reduce the air bubble penetration caused by water turbulent diffusion. For this case, it is suggested that *K*_a_ = *K*_w_/3. For fully aerated flows, the local *C*_a_ mainly exceeds 0.10 at the channel bottom when *C*_mean_ exceeds 0.40. The determinations of *K*_w_ and *D*_w_ are obtained from Eq. (33). The comparison between the measured data and Eq. (32) is shown in Fig. 4b. The good agreement indicates that homogenous water turbulent mixing in self-aerated open channel flows can predict the air concentration distribution for both high aerated regions (*C*_a_ > 0.5) and low aerated regions (*C*_a_ < 0.5). Under this situation, the sudden change in *y*_50_/*y*_90_ represents the difference in interior boundary variation and the capacity reduction of air diffusion for *C*_mean_ < 0.40. Because the ratio of *k*_1_/*k*_2_ = 1/3, *K*_w_ needs to be three times greater. The air turbulent diffusivity *K*_a_ can be set as *K*_a_ = *K*_w_/9 to obtain good agreement on the air concentration distribution in the low aerated region (*C*_a_ < 0.5). Consequently, the characterized flow depth *y*_50_, at which the local air concentration is 0.5, represents the interior transition boundary in self-aerated flows *C*_mean_ < 0.40, and the air–water mixture cross-section can be divided into water turbulent mixing and air turbulent mixing. For *C*_mean_ > 0.40, the full cross-section can be seen as the water mixing process, and the *y* < *y*_50_ region also fits the air turbulent mixing process.

**Figure 5:**
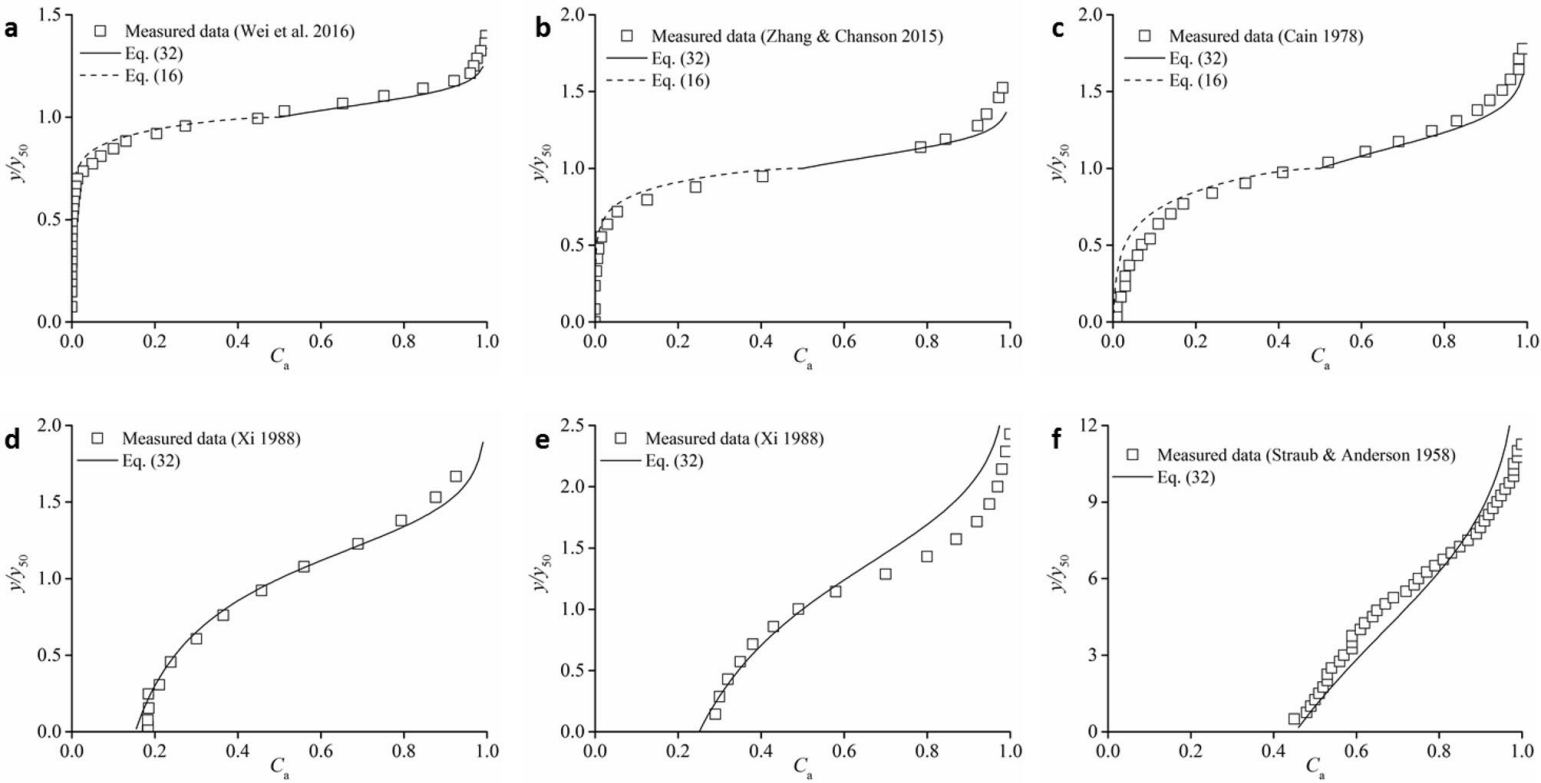
Comparison of calculated *C*_a_ profiles with the measured air concentration distribution in self-aerated open channel flows. (**a**) *C*_mean_ = 0.15 (smooth channel). (**b**) *C*_mean_ = 0.21 (stepped channel). (**c**) *C*_mean_ = 0.32 (smooth channel). (**d**) *C*_mean_ = 0.41 (smooth channel). (**e**) *C*_mean_ = 0.54 (smooth channel). (**f**) *C*_mean_ = 0.65 (smooth channel).

Based on Eqs. (16) and (32) with boundary conditions, the calculated air concentration profiles are compared with the experimental and prototype data^12,20,37–39^, as shown in Fig. 5. For each case, the determinations of *K*_w_ and *K*_a_ are deduced from each *C*_mean_ within the calibrated procedure proposed above. For *C*_mean_ < 0.40 within clear water, the air concentration distributions agree well for air and water turbulent mixing areas, and the theoretical results are applicable for both smooth and stepped spillways. Thus, it is reasonable to relate the diffusion behaviour of air turbulent mixing with water turbulent mixing. Air diffusion within the presence of clear water is verified to change *K*_a_ and restrain air diffusion according to the sudden change in the *y*_90_/*y*_50_ gradient for *C*_mean_ ≈ 0.40. Moreover, the air concentration profiles for high *C*_mean_ up to 0.65 agree with the theoretical results obtained from water turbulent mixing. This indicates that the local water turbulent mixing in the free-surface area plays an important role in the air–water mixture process. The air–water structures in the upper spray region, where macroscopic water roughness and microscopic water particles are predominant, are of various types, such as individual droplets, water projections, mixture clusters, and foam transported with the free surface. Owing to the individual water structures affected by the interaction between the free surface and the local turbulence, the satisfactory predicted results indicate that the movement of complex water-predominant structures can be simplified as local individual water particle movements affected by turbulence.

Most theoretical and observation descriptions of the aerated region of classical air–water mixtures in self-aerated flows involve a “layered” description: (1) individual water droplets in air in the top layer; (2) a mixture of water and air in the middle layer; and (3) individual air bubbles in water in the bottom layer. The layer is a generalization concept on the basis of the dominant air–water structures. In the present theoretical derivation process, there is no flow depth limit until the discussion of the satisfied condition for establishing the equation. The specific interior flow depth *y*_50_ is obtained because the boundary condition of particle concentration is 0.5 in the air or water mixing process affected by turbulent diffusion. The interior transition depth *y*_50_ is considered the transition layer from the top layer, where the water phase dominates, to the bottom layer, where the effect of the air phase increases. Consequently, the characterized water parcels and air parcels should be considered equally in high-speed chute flows, and the movement of complex air–water structures can be simplified as local particle turbulent diffusion. This promotes the further understanding and description of layer theory in self-aerated open channel flows.

## Self-aeration streamwise development

**Figure 6:**
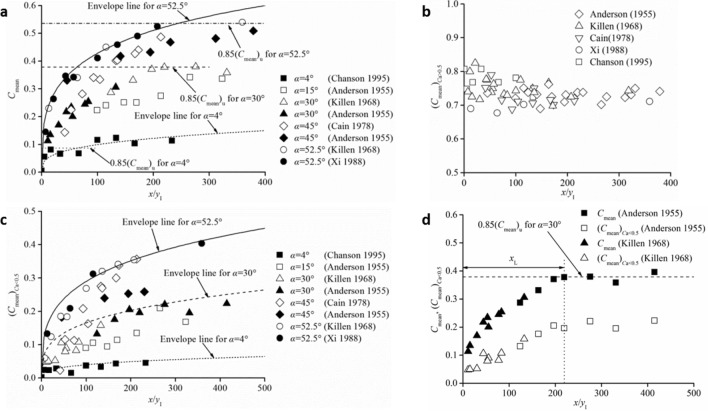
Typical self-aeration characteristics in the streamwise development process. (**a**) *C*_mean_. (**b**) (*C*_mean_)_*C*a>0.5_. (**c**) (*C*_mean_)_*C*a<0.5_. (**d**) Comparison of the developmental processes between *C*_mean_ and (*C*_mean_)_*C*a<0.5_.

When self-aeration of the free surface occurs in high-speed chute flows, the air–water mixes with an increase in the macroscopic *C*_mean_, as shown in Fig. 6a, where *x* is the distance from the self-aeration inception point along the streamwise direction, and *y*_I_ is the initial water flow depth at the inception point. *x*/*y*_I_ is used as the nondimensional parameter to illustrate the development of self-aerated flows. The water flow discharge per unit width ranges from 0.15 to 3.16 m^2^/s with *α* = 4°–52.5°^40–43^. Owing to the differences between water and air turbulent mixing areas, the mean cross-sectional air concentration can be divided into two parts demarcated by *y*_50_, separately defined as (*C*_mean_)_*C*a>0.5_ and (*C*_mean_)_*C*a<0.5_:

35 $$ (C_{{{\text{mean}}}} )_{{C{\text{a}} > {0}{\text{.5}}}} = \frac{1}{{y_{90} - y_{{{50}}} }}\int_{{y_{{{50}}} }}^{{y_{90} }} {C_{{\text{a}}} } {\text{d}}y,\quad y > y_{{{50}}} $$

36 $$ (C_{{{\text{mean}}}} )_{{C{\text{a}} < {0}{\text{.5}}}} = \frac{1}{{y_{50} }}\int_{0}^{{y_{50} }} {C_{{\text{a}}} } {\text{d}}y,\quad y < y_{{{50}}} $$

In Fig. 6b, the values of (*C*_mean_)_*C*a>0.5_ do not change notably in the entire development process, remaining at (*C*_mean_)_*C*a>0.5_ ≈ 0.75 in the incipient distance for 0 < *x*/*y*_I_ < 50 and slightly changing to (*C*_mean_)_*C*a>0.5_ ≈ 0.70 for *x*/*y*_I_ > 50. The development of (*C*_mean_)_*C*a<0.5_ is similar to the variation in *C*_mean_ as self-aeration develops downstream (Fig. 6c). This is because the air concentration in the upper free-surface area results from the growth of surface waves and entrapped air, which develops rapidly into small free-surface roughness and water droplets in the following incipient distance^4^. Altogether, once self-aeration occurs, the local water turbulent mixing affected by the free-surface turbulence is relatively stable. The development of an air–water mixture in a self-aerated open channel flow manifests mainly as air diffusion in the water. Compared with the two developments of *C*_mean_ and (*C*_mean_)_*C*a<0.5_ with other identical conditions in Fig. 6d, the approximately same trends confirm that as the air turbulent mixing approaches near full development, the entire air–water mixture of self-aerated flows reaches a uniform equilibrium level.

Furthermore, self-aeration typically reaches the near-fully developed region with an approximately identical development process under the same channel slope conditions, such as *α* = 30°, 45° and 52.5°, as shown in Fig. 6a and Fig. 6d. For uniform air–water mixture flow conditions, the mean cross-sectional air concentration (*C*_mean_)_u_ is dependent only on the channel slope. Hager^4^ analyzed the uniform (*C*_mean_)_u_ for 7.5° ≤ *α* ≤ 75° and defined it as

37 $$ (C_{{{\text{mean}}}} )_{{\text{u}}} = 0.75(\sin \alpha )^{0.75} $$

**Figure 7:**
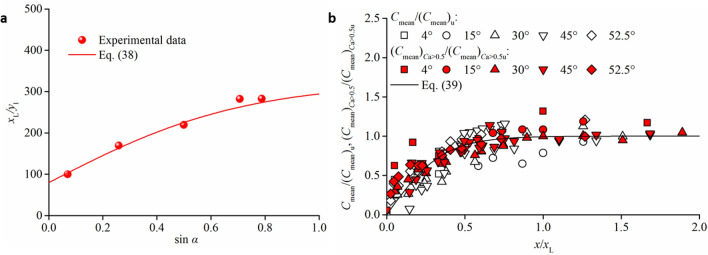
Relationships between self-aeration and development distance length. (**a**) effect of *α* on *x*_L_. (**b**) Variations in *C*_mean_ and (*C*_mean_)_*C*a<0.5_ in the self-aeration development distance.

This equation results in (*C*_mean_)_u_ being approximately 0.7 for a steep channel slope *α* = 75°, and it is in agreement with the *y*_50_/*y*_90_ variation within *C*_mean_. This indicates that *y*_50_/*y*_90_ decreases to zero for extremely developed aeration near *C*_mean_ = 0.7. In the present analysis, it is considered that the air–water flow reaches a near-fully developed region when *C*_mean_ ≥ 0.85(*C*_mean_)_u_ (reference lines in Fig. 6a), owing to differences in the experimental measurement. The streamwise distance length *x*_L_ from the inception point to the fully developed flow cross-section is defined as the self-aeration development length, as shown in Fig. 6d. For a specific channel slope condition, the average value is used, which is deduced from the experimental data. A larger channel slope corresponds to a longer distance of self-aeration development. In Fig. 7a, the relationship between *x*_L_/*y*_I_ and sin*α* can be expressed as

38 $$ \frac{{x_{{\text{L}}} }}{{y_{{\text{I}}} }} = 280 \cdot \tanh (1.2\sin \alpha ) + 80 $$

The developments of *C*_mean_ and (*C*_mean_)_*C*a<0.5_ can be applied as

39 $$ \frac{{C_{{{\text{mean}}}} }}{{(C_{{{\text{mean}}}} )_{{\text{u}}} }} = \frac{{(C_{{{\text{mean}}}} )_{{C{\text{a}} < 0.5}} }}{{(C_{{{\text{mean}}}} )_{{C{\text{a}} < 0.5{\text{u}}}} }} = \tanh \left( {2.8\frac{x}{{x_{{\text{L}}} }}} \right) $$

where (*C*_mean_)_*C*a<0.5u_ is the mean cross-sectional air concentration in the air turbulent mixing area (*y* < *y*_50_) for the fully developed uniform region. The streamwise normalization of Eq. (39) includes *x*/*x*_L_. Figure 7b compares all datasets with the data pertaining to Eq. (39), resulting in a coefficient of determination *R*^2^ = 0.847. The data trend indicates that self-aeration in high-speed chute flows develops rapidly in the incipient half of the streamwise distance length *x*/*x*_L_ < 0.5 and reduces gradually until the air–water uniform condition is reached. As Eqs. (38) and (39) describe *C*_mean_ using *y*_I_ and these relationships in self-aerated chute flows can be estimated for a given initial configuration, it is expected that the quantitative expressions are comparable for both model and prototype applications.

The present study proposes an analysis of air–water turbulent diffusion based on a theoretical model. Recent experimental and theoretical analyses confirm that a reasonable model for particle groups in air–water flows can result in reliable two-phase properties, including time-average mixture-velocity and air concentration distributions^44^. According to the present analysis results, the air–water mixture in high-speed chute flows is a result of the interaction of air and water under turbulence. Microscopic water and air turbulent mixing through the free surface plays an important role in air–water flow development. Further research on self-aeration onset should consider the formation of water ejection and air entrainment equally. The detailed differences in water ejection and air entrainment are required for a complete understanding of air–water mixture development, thereby helping promote hydraulic and environmental applications. Moreover, the effects of individual particle distribution and movement in different flow conditions, including flow turbulence intensity and mixture levels, should be further studied. The limitations and explanations should be clarified, which can contribute to the relation of air–water mixtures and a continuous phase analysis model.

## Data Availability

The raw data are available on request from the corresponding authors.
